# Balancing CIK Cell Cancer Immunotherapy and PPAR Ligands: One Potential Therapeutic Application for CNS Malignancies

**DOI:** 10.1002/cam4.70497

**Published:** 2024-12-16

**Authors:** Kira Vordermark, JingJing Pu, Amit Sharma, Jarek Maciacyzk, Ingo G. H. Schmidt‐Wolf

**Affiliations:** ^1^ Department of Integrated Oncology, Center for Integrated Oncology (CIO) University Hospital of Bonn Bonn Germany; ^2^ Department of Stereotactic and Functional Neurosurgery University Hospital of Bonn Bonn Germany

**Keywords:** cytokine‐induced killer cells, glioblastoma, neuroblastoma, peroxisome proliferator‐activated receptors, WNT/β‐catenin

## Abstract

**Background:**

Cytokine‐induced killer (CIK) cell therapy has proven successful in clinical trials regarding glioblastoma. Equally important are the hints suggesting peroxisome proliferator‐activated receptors (PPARs) ligands being co‐expressed in the central nervous system (CNS). This provides a rationale about investigating the possible synergistic effect of CIK cells and PPARs.

**Methodology:**

We investigated neuroblastoma and glioblastoma cell lines with mature CIK cells and the PPARγ antagonist GW‐9662 to assess the effects on cell viability, candidate gene expression (Wnt/β‐catenin signalling, DNMT1) and global methylation levels (5‐methylcytosine, LINE‐1).

**Results:**

Using a clinical applicable PPAR‐γ inhibitor, we showed that (1) PPARγ‐antagonist GW‐9662 suppressed tumor cell growth in both neuroblastoma and glioblastoma cells, (2) PPARγ inhibition had restricted effect on the expression of Wnt/β‐catenin associated genes, (3) inhibition of PPARγ led to downregulation of DNMT1 expression, supporting their hypothesized interaction in cancer, (4) a partial modulation of global LINE‐1 methylation levels was observed, indicating their role in epigenetic processes, and (5) Combining PPARγ inhibition with CIK cell immunotherapy enhanced cell lysis significantly.

**Conclusion:**

We provide evidence that PPAR ligands in combination with CIK cell immunotherapy could be a valuable option for malignant CNS tumors.

## Graphical abstract

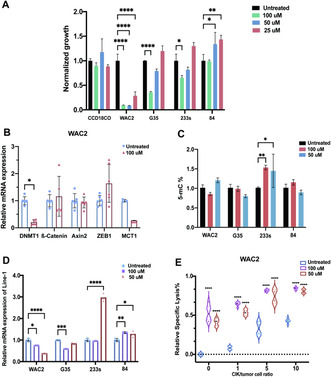

The fundamental idea of using the body's own immune system to combat cancer has proven to be extremely successful in adoptive cancer immunotherapy. In this context, CIK cell therapy has revolutionized the field of oncology by prolonging the survival of patients afflicted with fatal cancers [1]. Recently, a successful clinical trial involving CIK cells in patients with pathologically pure glioblastoma [2], has now opened an avenue for its potential application in CNS malignancies. While experience across various cancers implies that CIK cells are compatible with a wide range of immune checkpoint inhibitors, epigenetic drugs, or commercial agents [3], a potential synergistic effect when combined with peroxisomal proliferator‐activated receptors (PPARs) has not yet been investigated. Particularly in the context of CNS diseases, several studies have supported the hypothesis of using PPARγ agonists in neuroblastoma and glioblastoma [4, 5, 6, 7]. Beyond that, the potential effect of PPAR‐γ in brain tumors has also been investigated in some clinical studies [8, 9]. Therefore, it is reasonable to investigate the synergetic effect of PPAR‐γ and CIK cells as a possible treatment strategy for CNS malignancies.

Considering this, we asked (1), whether a clinically applicable PPARγ antagonist (GW‐9662) would suppress tumor cell growth in neuroblastoma and glioblastoma cells, and (2), whether it would affect the expression of genes associated with Wnt/β‐catenin which are known to play an important role in GBM pathogenesis (Figure 1, Figure S1). We first confirmed that GW‐9662 suppresses tumor cell growth in both neuroblastoma (WAC2) and glioblastoma cell lines (G35, BTS233, and 84) (Figure 1A).

**FIGURE 1:**
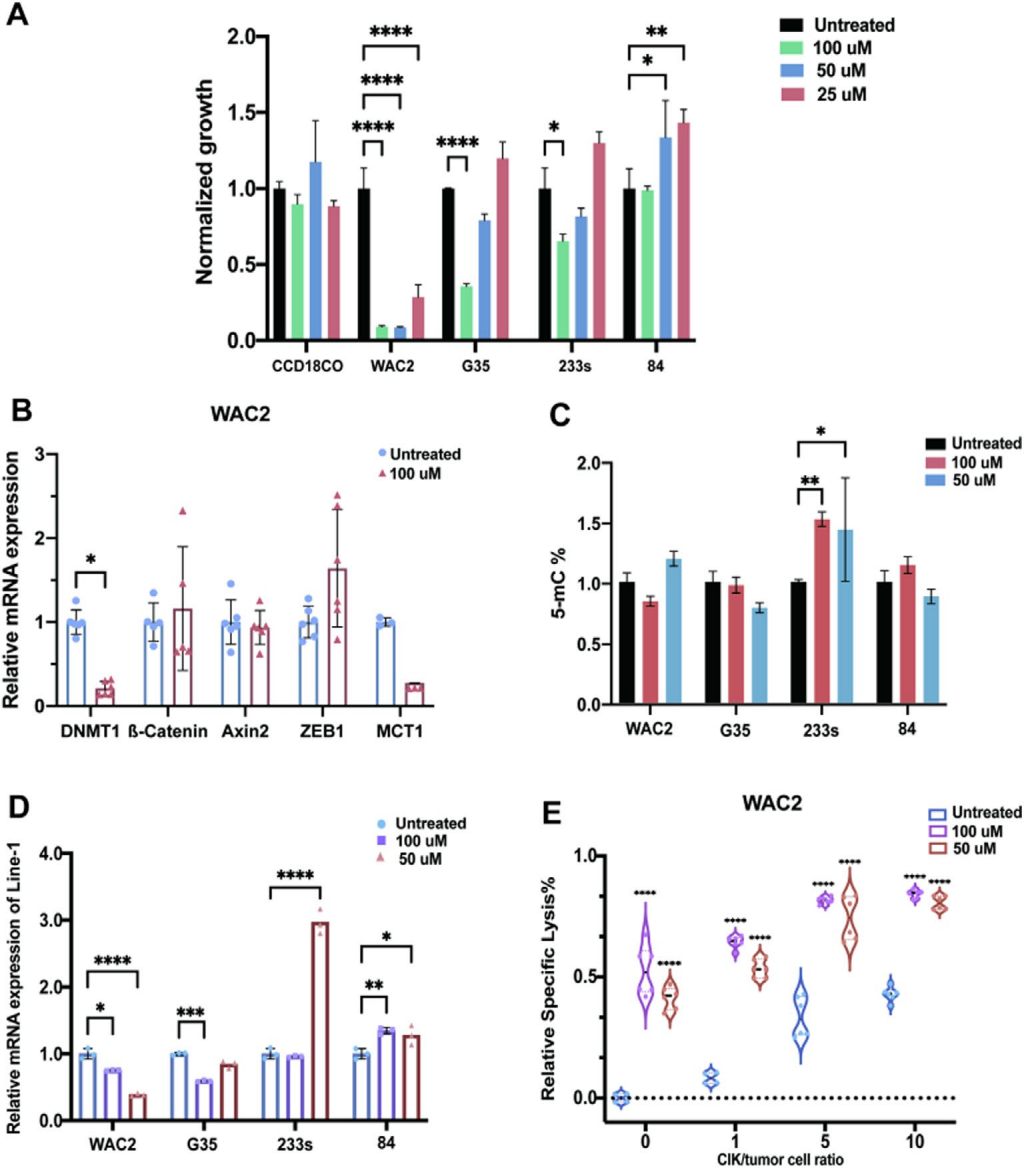
(A) Cell growth was measured on the y‐axis using a CCK‐8 kit and normalized to DMSO, after treating with GW‐9662 for 72 h. On the *x*‐axis, the control cell line CCD18CO is on the far left, followed by glioblastoma (G35, 233s, and 84) and neuroblastoma (WAC2) cell lines, arranged from higher to lower growth. (B) The expression levels of DNMT1, β‐catenin, and their target genes (Axin2, ZEB1, and MCT) in the Wnt/β‐catenin pathway were measured in WAC2 cells using qPCR after 72 h of treatment with 100 μM GW‐9662, normalized to DMSO. (C) The percentage of 5‐methyl‐cytosine was measured using an ELISA assay and shown on the y‐axis, but the results did not match the reduced methylation observed in LINE‐1 qPCR. Changes in LINE‐1 mRNA levels after treatment with GW‐9662 at concentrations of 100/50 μM/DMSO were charted across the WAC2, G35, 233s, and 84 cell lines. Significant mRNA reduction was noted in WAC2 at both concentrations and in G35 at 100 μM only. (D) GW‐9662 treatment significantly reduced LINE‐1 mRNA expression in two out of four cell lines, particularly in WAC2 at both tested concentrations and in G35 at 100 μM only. (E) Treatment of cell line WAC2 with both CIK cells at CIK‐to‐tumor‐cell‐ratios of 0/1/5/10 and GW‐9662 at 100/50 μM yielded mostly significant increases in relative specific lysis compared to DMSO treatment only. The results represent data from three separate experiments and are presented as mean ± SD. Significance levels were determined using two‐way ANOVA with Bonferroni's post hoc test (*p < 0.05, **p < 0.01, ***p < 0.001, ****p < 0.0001).

Because the Wnt/β‐catenin signaling pathway is involved in multiple stages of neuroblastoma development and promotes GBM development and progression [10, 11], we next investigated the expression levels of genes associated with Wnt/β‐catenin signaling such as Axin2, ZEB1, and MCT. We found significant upregulation of Axin 2 in GBM cell lines (G35, 84; *p* ≤ 0.001). In addition, we focused on the possible interactions between PPARγ and DNMTs (DNA methyltransferase) which has recently been discussed to play a yet to be defined role in cancer [12]. In context to the expression level of DNMT1, we observed significant downregulation in neuroblastoma cells after GW‐9662 treatment (*p* ≤ 0.05) (Figure 1B). We further quantified 5‐methylcytosine levels using ELISA and detected an upregulation thereof in GBM cells (BTSC233; *p* ≤ 0.01 for 100 μM and *p* ≤ 0.05 for 50 μM, respectively) (Figure 1C). The findings on global methylation levels also prompted us to determine any possible alterations in the methylation level of Long‐Interspersed Nucleotide Element‐1 (LINE‐1), a surrogate marker for global methylation. Of interest, we verified that PPARγ inhibition significantly reduced LINE‐1 expression in WAC2 (*p* ≤ 0.05 for 100 μM, *p* ≤ 0.01 for 50 μM) G35 (*p* ≤ 0.0001 for 50 μM) (Figure 1D).

Next, we investigated the compatibility of GW‐9662 with the CIK cell adoptive immunotherapy approach. Using variable concentrations (E:T ratios) of GW‐9662 with CIK cells, we found that the CIK cells exert cytotoxic effect in both glioblastoma and neuroblastoma cells which is further enhanced significantly owing to the synergistic effect of the PPARγ inhibitor (Figure 1E, *p* ≤ 0.0001). Having found that DNMT1 expression was altered following PPARγ inhibition, we also tested whether the DNMT1 inhibitor (5‐azacytidine, 5‐aza) could have a direct effect on CIK cells. To determine this, we used different concentrations (E:T ratios) of DNMT inhibitor with CIK cells and found that a high DNMT1 concentration was required to significantly increase the cytotoxic capacity of CIK cells in neuroblastoma cells (Figure S1). The detailed description of the methodology is included in Appendix S1.

In this proof of principle study, we have shown that a clinically applicable PPARγ antagonist (GW‐9662) has inhibitory effects on neuroblastoma and glioblastoma cell lines. Though, the effect was not pronounced in the regulation of key genes associated with Wnt/β‐catenin signaling, still AXIN2 appeared to be affected in GBM cells. Here, it is noteworthy to mention about the changes we observed in components of the epigenetic machinery (DNMT1 and LINE‐1). In addition, the general compatibility of CIK cells with PPARγ and the restrictive effect of DNMT1 should be mentioned. Undeniably, a further confirmation of our findings in in vivo model could facilitate the clinical translation of PPARγ‐CIK cells combo in the clinical spectrum of CNS malignancies.

## Author Contributions

**Kira Vordermark:** methodology (equal), writing – original draft (lead), writing – review and editing (equal). **JingJing Pu:** methodology (equal), writing – review and editing (equal). **Amit Sharma:** conceptualization (equal), writing – review and editing (equal). **Jarek Maciacyzk:** conceptualization (equal), writing – review and editing (equal). **Ingo G. H. Schmidt‐Wolf:** conceptualization (equal), writing – review and editing (equal).

## Ethics Statement

The authors have nothing to report.

## Consent

The authors have nothing to report.

## Conflicts of Interest

The authors declare no conflicts of interest.

## Supporting information


**Figure S1.** (A) Relative expression levels of DNMT1 and β‐catenin and associated genes (Axin2, ZEB1, and MCT) were determined in G35, 233s, and 84 after 72 h of treatment with 100 μM GW‐9662 by qPCR. (B) Cell lysis induced by a combination of GW‐9662 with cytokine‐induced killer (CIK) cells in glioblastoma cell lines G35, 233s, and 84 and control cell line CCD180CO is shown. Variable CIK‐to‐tumor‐cell‐ratios (0, 1, 5, and 10) were used with GW‐9662 (100 and 50 μM). The results represent data from three separate experiments and are presented as mean ± SD. Significance levels were determined using two‐way ANOVA with Bonferroni’s post hoc test (*p < 0.05, **p < 0.01, ***p < 0.001, and ****p < 0.0001).

**Appendix S1.** The detailed description of the material and method used in the study.

## Data Availability

The authors have nothing to report.
