# Supplementary figures and images for: Balancing CIK Cell Cancer Immunotherapy and PPAR Ligands: One Potential Therapeutic Application for CNS Malignancies
Source: Cancer Med. 2024 Dec 16;13(24):e70497. doi: 10.1002/cam4.70497 (PMC11647548; doi:10.1002/cam4.70497)

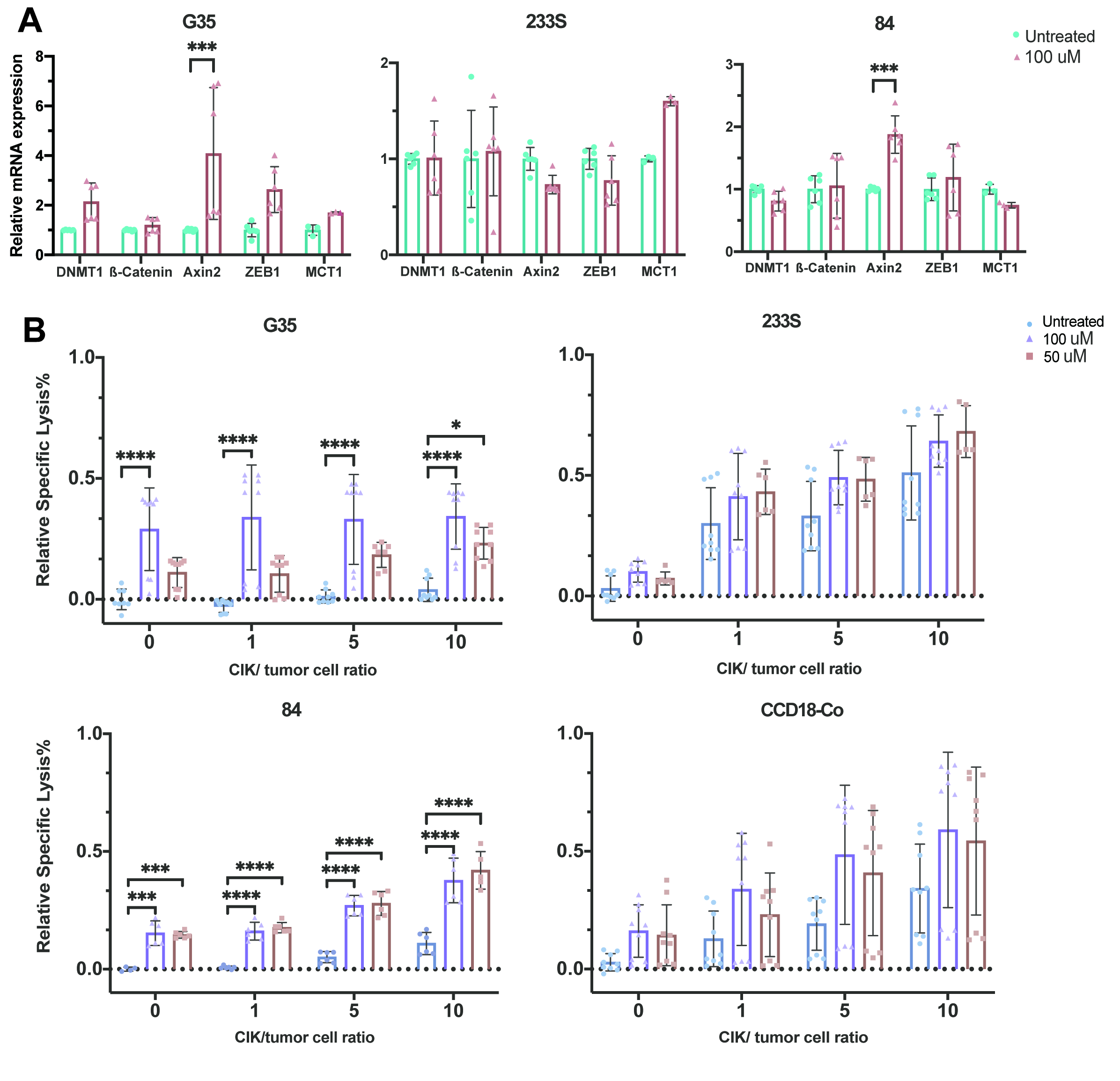

Supplement: Supplementary file 1 — Figure S1. (A) Relative expression levels of DNMT1 and β‐catenin and associated genes (Axin2, ZEB1, and MCT) were determined in G35, 233s, and 84 after 72 h of treatment with 100 μM GW‐9662 by qPCR. (B) Cell lysis induced by a combination of GW‐9662 with cytokine‐induced killer (CIK) cells in glioblastoma cell lines G35, 233s, and 84 and control cell line CCD180CO is shown. Variable CIK‐to‐tumor‐cell‐ratios (0, 1, 5, and 10) were used with GW‐9662 (100 and 50 μM). The results represent data from three separate experiments and are presented as mean ± SD. Significance levels were determined using two‐way ANOVA with Bonferroni’s post hoc test (*p < 0.05, **p < 0.01, ***p < 0.001, and ****p < 0.0001). [file CAM4-13-e70497-s001.png]
