# Supplementary material for: Balancing CIK Cell Cancer Immunotherapy and PPAR Ligands: One Potential Therapeutic Application for CNS Malignancies
Source: Cancer Med. 2024 Dec 16;13(24):e70497. doi: 10.1002/cam4.70497 (PMC11647548; doi:10.1002/cam4.70497)
Supplement: Supplementary file 2 — Appendix S1. The detailed description of the material and method used in the study. [file CAM4-13-e70497-s002.docx]

**Supplementary file 1:**

**Materials and methods**

**Cell culture**

We utilized one human neuroblastoma cell line (WAC2) and three human glioblastoma cell lines (G35, 84, 233s) in this study all of which were maintained at 37 degrees Celsius with 5% CO_2_. WAC2, G35, 84, 233s were cultured adherently for cell viability, flow cytometry, and RNA extraction assays in DMEM (Gibco, Grand Island, NY, USA), supplemented with 10% heat-inactivated fetal bovine serum (FBS, Gibco, Grand Island, NY, USA), 100 U / ml penicillin and 100 µg / ml streptomycin (Gibco, Grand Island, NY, USA). All cell lines were routinely tested for Mycoplasm with MycoAltert mycoplasma detection kit (Lonza, Basel, Switzerland).

CCD18CO, initially isolated from normal colon tissue and exhibiting fibroblast morphology, was used as a control cell line, and cultured in MEM media (Gibco, Grand Island, NY, USA), supplemented with 10% fetal bovine serum, 100 U / ml penicillin and 100 µg / ml streptomycin (Gibco, Grand Island, NY, USA).

The selective PPARγ antagonist GW-9662 (2-Chloro-5-nitrobenzanilide) was purchased from Focus Biomolecules (Plymouth Meeting, PA, USA).

**Cell Counting Kit-8 (CCK-8) Assay**

A CCK-8 kit (Dojindo Laboratories, Inc., Kumamato, Japan) was used to evaluate cell viability. On day 1, 3 x 10^5^ cells/well were plated in 96-well plates. On day 2, when the cells had attached to the plate during an overnight incubation, media was aspirated and replaced with 100 μl media containing GW-9662 at a final concentration of 100 μM / 50 μM/ 25 μM or DMSO. After incubation for 72 hours with the compound / vehicle, on day 5, in accordance with the manufacturer´s protocol, 10 μl of CCK-8 solution were added per well and absorbance was measured at 450 nm and 600 nm. OD values at 600 nm were subtracted from those at 450 nm, blank / media only wells were subtracted from all wells, then normalized to DMSO treated wells. Experiments were performed in triplicate.

**Generation of cytokine-induced killer cells**

Peripheral blood mononuclear cells were obtained from buffy coats of three independent healthy donors by gradient density centrifugation as previously described (1). After sequential incubation with IFN-γ and IL-1β and IL-2, CIK cells were expanded in vitro for 14 days after which they were considered mature. Medium was changed every 3-4 days during maturation.

**In vitro cytotoxicity analysis by flow cytometry**

Briefly, 2× 10^6^ target cells were labeled with 5 μM fluorescent carboxyfluorescein succinimidyl ester (CFSE, Thermo Fisher Scientific, Eugene, USA) cell dye in 1 ml PBS (PAN-Biotech, Aidenbach, Germany) for 25 min at 37°C in the dark, followed by three washes with 5 ml of culture medium which contained 10 % FBS to quench the any CFSE reaction.

Labelled target cells were incubated with GW-9662 and CIK cells, at specified effector-to-target (E:T) ratios (1:1, 5:1 and 10:1), for 24 hours each. 10,000 CFSE-positive events were recorded, all experiments were performed in triplicate. Cell viability was analyzed by flow cytometry following staining with viability dye Hoechst 33258 (final concentration 0.5 μg / ml; Cayman Chemical, Hamburg, Germany) without further washing and incubation. The samples were analyzed immediately using a FACSCanto II Flow Cytometer (BD Biosciences, Heidelberg, Germany). Relative specific lysis was calculated as previously described by Wu et al. (2).

Specific lysis (%) = ((TC-TE) / TC) * 100,

TC representing the percentage of live CFSE^+^ target cells in control tubes and TE representing the percentage of live CFSE^+^ target cells in test tubes (target cells + effector cells).

Quantitative analysis performed was in FlowJo v10.6 software (FlowJo, LLC, Ashland, OR, USA).

**Real-time reverse transcription-qPCR (RT-qPCR)**

WAC2, G35, 84 and 233s were replated at a confluency of 500,000 cells / well on 6-well plates. After 24 hours, cells were treated with vehicle (DMSO) or GW-9662 at 50 / 100 μM. Total RNA was extracted using RNeasy Mini Kit (Qiagen, Hilden, Germany) according to the manufacturer´s instructions.

cDNA amplification was performed using High-Capacity cDNA Reverse Transcription Kit (Applied Biosystems, Foster City, CA, Ref. 4368813) according to the manufacturer´s protocol. 1000 ng of extracted RNA were added to 1 μl of reverse transcriptase and, in a final volume of 20 μl, at a final concentration of 1X, 2 μl reverse transcriptase buffer, 2 μl random primers and 0.8 μl dNTPS.

Quantitative RT-PCR analysis was performed on an ABI Real Time PCR System (Applied Biosystems, Foster City, CA), (95C, 15 sec + 60C, 60 sec) x 40 cycles, using PowerTrack SYBR Green Master Mix (Applied Biosystems,). A final volume of 20 μl per single qPCR reaction contained 10 μl PowerTrack SYBR Green Master Mix, resulting in a final concentration of 1X, 2 μl of a 1:1 mix of forward and reverse primers at a final concentration of 300 nM (stock concentration: 3 μM) and 8 μl of nuclease-free water. The incorporation of SYBR Green into the PCR products was monitored in real time after each PCR cycle. Respective C_t_ values defining the PCR cycle number at which exponential growth of PCR product exceeds the threshold were calculated.

The sequences for primer used to amplify mRNA are as follows. GAPDH forward, 5´-GCACCGTCAAGGCTGAGAAC-3´, reverse, 5´-TGGTGAAGACGCCAGTGGA-3´; PPAR gamma forward, 5´-TGG GGT TCT CAT ATC CGA GGG-3´, reverse, 5´-TCA CAT TCA GCA AAC CTG GGC-3´; DNMT1 forward, 5´-CCTAGCCCCAGGATTACAAGG-3´, reverse 5´-ACTCATCCGATTTGGCTCTTTC-3´; ß-catenin forward, 5´-ACAGCAGCAATTTGTGGAGGG-3´, reverse, 5´-AGCCAAGTTCACAGAGGACCC-3´; LINE-1 forward, 5´-GTA CCG GGT TCA TCT CAC TAG G-3´, reverse, 5´-TGT GGG ATA TAG TCT CGT GGT G-3´; TATA-binding protein forward, 5´-ACA ACA GCC TGC CAC CTT A-3´, reverse, 5´-GAA TAG GCT GTG GGG TCA GT-3´.

Normalization was executed on the housekeeping gene (GAPDH or TATA, respectively) and calculated according to the ΔΔCt algorithm (3). Results are expressed as means (n = 3) ± standard deviation, one-fold expression representing the control.

**LINE-1 DNA methylation assay**

DNA was extracted from 250,000 cells / well using Qiagen´s Blood & Cell Culture DNA Mini Kit (Ref 13323) and purified using Genomic DNA Buffer Set (Ref 19060, both Qiagen, Hilden, Germany) according to the manufacturer´s instruction. 500 ng of the extracted DNA was used for MseI digestion, and 100 ng of the digested DNA was subsequently placed in each well (triplicate).

Using the Global DNA Methylation Assay enzyme-linked immunosorbent assay (ELISA) system (Active Motif, Carlsbad, California, USA), 5-mC DNA was quantified as specified by the manufacturer. Absorption was measured at 450 nm and at reference wavelength of 655 nm, blank wells were subtracted, and absorption at reference wavelength was subtracted. To calculate the 5%-mC associated with the detectable CpG residues in the samples, a standard curve using standards of 0/5/10/20/30/50/75/100 % 5-mC was generated.

**Statistical analysis**

Statistical analyses were performed using GraphPad Prism v.9.0 (GraphPad Software, Inc., San Diego, CA, USA). One-way or two-way analysis of variance (ANOVA) with Dunnett´s or Tukey's multiple comparison test, respectively, was performed to analyze statistical significance between groups. When calculating statistical significance to a p-value < 0.05, the statistical significance is illustrated by *, whereas ** represents p < 0.01, *** represents p < 0.001, and **** represents p < 0.0001. All results are presented as means ± standard deviation.

**References**

1. Schmidt-Wolf, I. G., Negrin, R. S., Kiem, H. P., Blume, K. G. & Weissman, I. L. 1991 Use of a SCID mouse/human lymphoma model to evaluate cytokine-induced killer cells with potent antitumor cell activity. J Exp Med 174, 139–149.

2. Wu, X., Zhang, Y., Li, Y. & Schmidt-Wolf, I. G. H. 2021 Improvements in Flow Cytometry-Based Cytotoxicity Assay. Cytometry A 99, 680–688.

3. Livak, K. J. & Schmittgen, T. D. 2001 Analysis of relative gene expression data using real-time quantitative PCR and the 2(-Delta Delta C(T)) Method. Methods 25, 402–408.
